# The Increased Dissolution and Oral Absorption of Itraconazole by Nanocrystals with an Endogenous Small-Molecule Surfactant as a Stabilizer

**DOI:** 10.3390/molecules29081769

**Published:** 2024-04-13

**Authors:** Sheng Chang, Qiang Yang, Jiahuan Liu, Li Yin, Jihong Han, Lanlan Zong, Xiaohui Pu

**Affiliations:** 1 College of Pharmacy, Jilin Medical University, Jilin 132013, China; 2 State Key Laboratory of Antiviral Drugs, School of Pharmacy, Henan University, Kaifeng 475004, Chinalanlan198903@126.com (L.Z.); 3 School of Pharmacy and Bioengineering, Keele University, Kiel ST5 5BG, UK; j.han@keele.ac.uk

**Keywords:** itraconazole, nanocrystals, cholic acid, supersaturation dissolution, in vivo pharmacokinetics, bioavailability

## Abstract

The aim of this study was to develop cholic-acid-stabilized itraconazole nanosuspensions (ITZ-Nanos) with the objective of enhancing drug dissolution and oral absorption. A laboratory-scale microprecipitation–high-pressure homogenization method was employed for the preparation of the ITZ-Nanos, while dynamic light scattering, transmission electron microscope analysis, X-ray diffraction, differential scanning calorimetry, and high-performance liquid chromatography analysis were utilized to evaluate their physicochemical properties. The absorption and bioavailability of the ITZ-Nanos were assessed using Caco-2 cells and rats, with Sporanox^®^ pellets as a comparison. Prior to lyophilization, the particle size of the ITZ-Nanos measured approximately 225.7 nm. Both X-ray diffraction and differential scanning calorimetry confirmed that the ITZ remained crystalline within the nanocrystals. Compared to the pellets, the ITZ-Nanos exhibited significantly higher levels of supersaturation dissolution and demonstrated enhanced drug uptake by the Caco-2 cells. The AUC(0–t) value for the ITZ-Nanos in rats was 1.33-fold higher than that observed for the pellets. These findings suggest that cholic acid holds promise as a stabilizer for ITZ nanocrystals, as well as potentially other nanocrystals.

## 1. Introduction

Pathogenic fungi encompass a diverse group of microorganisms that can induce various diseases in humans, animals, and plants [1]. Fungal infections have a considerable prevalence and pose a significant public health concern. Certain fungal pathogens have the potential to cause severe infections with high mortality rates. For instance, invasive candidiasis caused by *Candida tropicalis* infections has been associated with mortality rates ranging from 55% to 60% in adults and 26% to 40% in children. Additionally, *Histoplasma* spp. infections may result in mortality rates ranging from 21% to 53% in patients with Human Immunodeficiency Virus (HIV) and Acquired Immune Deficiency Syndrome (AIDS) [2]. The treatment of fungal infections remains a significant challenge, with itraconazole (ITZ) being one of the medications recommended by the World Health Organization (WHO) for this purpose.

ITZ is classified as an azole antifungal and antiparasitic agent with a wide range of activity, making it suitable for treating several high-priority fungal pathogens, including *Candida tropicalis*, *Cryptococcus gattii*, *Scedosporium* spp., and *Coccidioides* spp. Despite its efficacy, ITZ exhibits poor aqueous solubility levels of approximately 1 ng/mL at a neutral pH and 4 μg/mL at an acidic pH, placing it within the biopharmaceutics classification system class II (BCS II) [3,4]. Enhanced solubility and an enhanced dissolution rate are crucial factors for improving the oral bioavailability of BCS II drugs, as dissolution is a rate-limiting factor. According to the Ostwald–Freundlich equation, when the particle size is reduced to the nanometer range, the total surface area and saturation solubility of the drug particles will increase significantly [5]. Furthermore, drug particles in the nanometer range change the cellular uptake pathway of the drug in the intestines to increase its bioavailability compared to crude drugs. [6,7]. In recent years, various strategies have been investigated to enhance the aqueous solubility and dissolution rate of ITZ, including self-emulsifying capsules [8], engineered particle compositions [9,10], amorphous solid dispersions [11,12,13,14,15], nanosuspensions, and nanocrystals [16,17,18,19,20]. In addition to the two commercially available forms of ITZ, namely capsules and oral solutions [14,21], despite extensive efforts aimed at enhancing its solubility and bioavailability, developing an oral dosage form with improved bioavailability remains a formidable challenge.

Among the various strategies explored, nanocrystals are promising because of their high loading capacity and potential to enhance dissolution and bioavailability [22]. However, a previous report showed that improved dissolution with nanocrystals does not necessarily result in improved bioavailability [23]. Therefore, it is desirable to develop nanocrystals with different stabilisers that can effectively enhance both their dissolution and bioavailability.

Cholic acid (CA), an endogenous small molecule secreted in mammalian bile, plays a pivotal role as a physiological cleanser by efficiently solubilizing dietary fats, fat-soluble vitamins, and cholesterol [24] Due to its ready availability, non-toxic nature, and amphiphilic properties, which facilitate easy adsorption onto hydrophobic particles [25], it plays a crucial role in maintaining metabolic homeostasis as an essential component. Moreover, CA exhibits antifungal properties [26] and has been demonstrated to enhance the oral absorption of Sporanox^®^ pellets encapsulated in capsules [8,21], which makes it more attractive as a stabilizer for ITZ nanocrystals. In this study, CA was selected as a stabilizer, and a combination of microprecipitation and high-pressure homogenization methods was employed to prepare the nanocrystals of itraconazole (ITZ-Nanos). Initially, the impact of the drug–stabilizer ratio on the particle size was investigated, followed by an evaluation of various pharmaceutical properties of the ITZ-Nanos, including particle size, crystal type, morphology, and in vitro dissolution. Furthermore, stability assessments were conducted for the freeze-dried ITZ-Nanos and reconstituted liquid dispersions under storage conditions. Finally, in vitro cellular uptake experiments and in vivo pharmacokinetic studies were performed to assess the oral bioavailability of the ITZ-Nanos compared to Sporanox^®^ pellets.

## 2. Results and Discussion

### 2.1. Preparation and Characterization of the ITZ-Nanos

**Figure 1:**
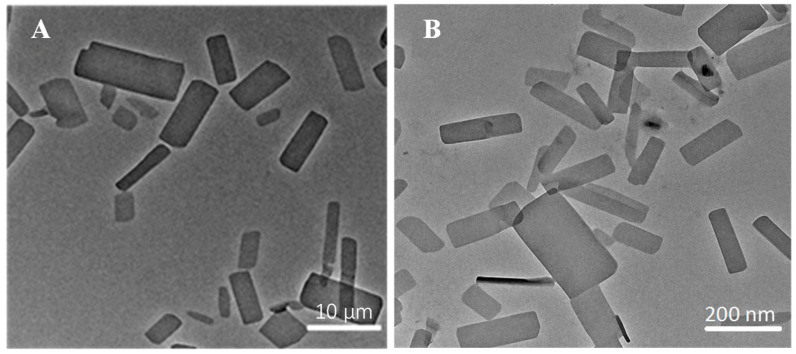
The morphology of bulk ITZ (**A**) and ITZ-Nanos (**B**) observed under TEM.

The ITZ-Nanos were prepared at different drug-to-stabilizer ratios (CA), and their particle sizes were measured. The results are presented in Table 1. It is evident that the ITZ-Nanos with a drug-to-stabilizer ratio of 1:1 exhibited the smallest particle size (225.7 nm), which was approximately 50 nm smaller than that of the ratio 2:1 and about 34 nm smaller than that of the ratio 1:2. Moreover, the ITZ-Nanos at a drug-to-stabilizer ratio of 1:1 showed a significantly lower polydispersity index (PDI) and a higher zeta potential compared to those at ratios of 2:1 and 1:2, indicating a more homogeneous particle size distribution and strong electrostatic repulsion for the samples with a ratio of 1:1. As is well established, an adequate amount of stabilizer is crucial to effectively cover the surface area of the drug particles and establish steric repulsion to achieve nanocrystal stability [27]. The insufficient stabilizer in the sample with a drug-to-stabilizer ratio of 2:1 may have contributed to the observed larger particle size (273 nm) and PDI (0.288) than that of the 1:1 ratio. However, decreasing the drug-to-stabilizer ratio from 1:1 to 1:2 did not further improve the formulation. Instead, it resulted in a larger particle size (262.1 nm) and PDI (0.268), suggesting that an excess amount of stabilizer might actually provide less stabilizing effect, a phenomenon also observed in separate studies utilizing different stabilizers [5]. This phenomenon could be interpreted as follows: When the stabilizer concentrations exceed the plateau of the adsorption isotherm, electrostatic stabilizers could reduce the diffuse layer, thereby leading to a decreased zeta potential and, consequently, decreased physical stability [28]. Therefore, optimizing the stabilizer concentration is widely acknowledged as crucial to the stabilization of nanoparticles in a drug/stabilizer system [29]. Our findings demonstrate that 1:1 proved to be the most effective among the tested ratios. Consequently, the ITZ-Nanos with a ratio of 1:1 were selected for further lyophilization. Notably, the mean particle size (242.7 nm) and zeta potential (−27.6 mV) of the lyophilized ITZ-Nanos remained comparable to those of the freshly prepared liquid ITZ-Nanos. These results indicate that the lyophilization process had a minimal impact on the particle size and zeta potential. The morphology of the interfacial transition zone (ITZ) observed under transmission electron microscopy (TEM) is depicted in Figure 1. In the ITZ-Nanos, the ITZ exhibited a rectangular shape resembling that of the bulk drug; however, the particle size of the ITZ-Nanos was significantly smaller, being tens of times reduced compared to its bulk counterpart. The observed particle size under TEM (Figure 1) was slightly smaller than that seen using dynamic light scattering (225.7 ± 10.3 nm), which was attributed to the fact that these particles were dried and their CA layer shrunk after losing water in the TEM experiments [30]. This reduction in particle size confers advantageous properties for enhancing the dissolution rate of ITZ by augmenting both its total surface area and saturation solubility [5].

### 2.2. Crystalline State of the ITZ-Nanos

**Figure 2:**
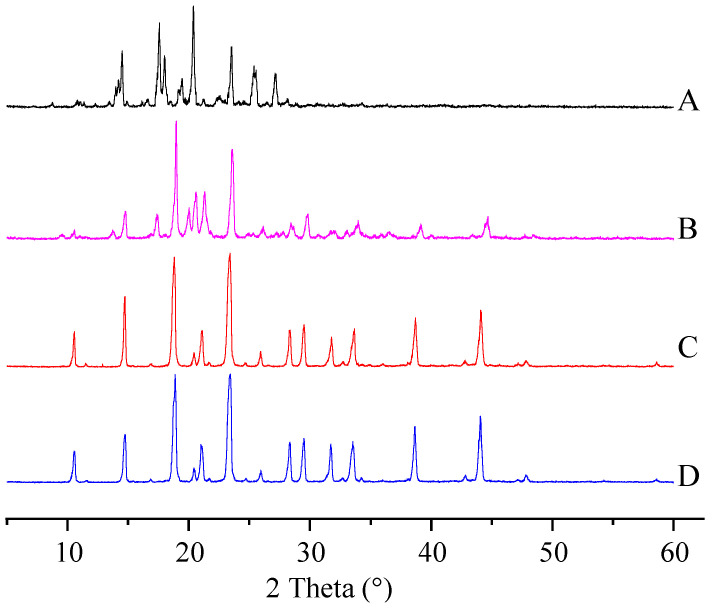
X-ray diffraction patterns of (**A**) the bulk ITZ; (**B**) lyophilized ITZ-Nanos; (**C**) blank excipients; (**D**) physical mixture of CA, mannitol, and ITZ.

**Figure 3:**
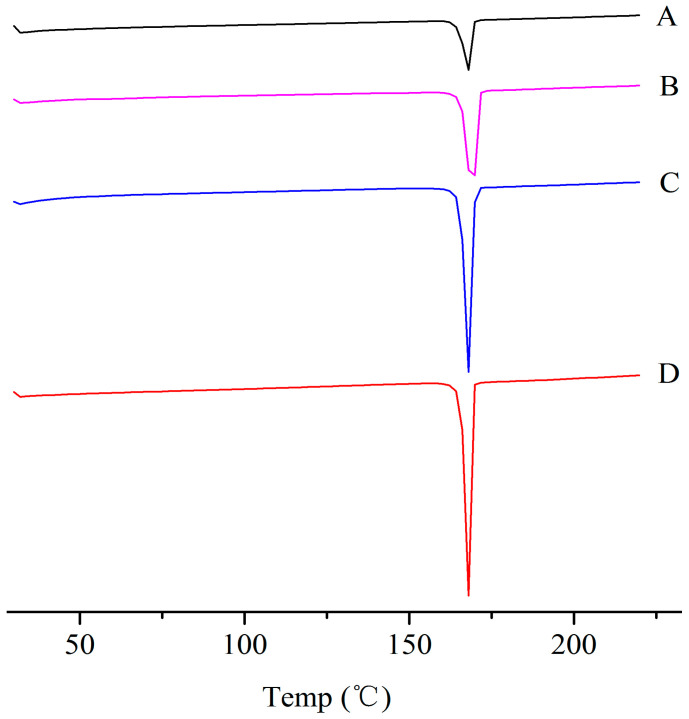
DSC diagrams of (**A**) the bulk ITZ; (**B**) lyophilized ITZ-Nanos; (**C**) blank excipients; (**D**) physical mixture of CA, mannitol, and ITZ.

The physical properties of the ITZ in the ITZ-Nanos were investigated using differential scanning calorimetry (DSC) and X-ray diffraction. Figure 2 shows the XRD patterns of the pure ITZ, the blank excipients (physical mixtures of CA and mannitol), a physical mixture of CA, mannitol and ITZ, and the lyophilized ITZ-Nanos. The diffraction patterns of the crude ITZ and the lyophilized ITZ-Nano powder showed characteristic diffraction peaks between 10° and 30°, suggesting that the lyophilized powder remained crystalline. The diffraction patterns of the blank excipients and the physical mixture of CA, mannitol, and ITZ were nearly identical, which was mainly due to the cholic acid and mannitol. The contribution of the ITZ to the physical mixture was negligible, probably due to the low percentage of ITZ in the mixture, which was easily submerged by the peaks of CA and mannitol. The DCS results for the bulk ITZ, the blank excipients, the physical mixture of CA, mannitol and ITZ, and the lyophilized ITZ-Nanos are shown in Figure 3. Both the pure ITZ and the lyophilized ITZ-Nanos exhibited a sharp melting process with two endothermic peaks at 168.4 °C and 168.22 °C, respectively, suggesting that the ITZ in the lyophilized ITZ-Nanos remained crystalline, consistent with the XRD results. The endothermic peaks of the blank excipients and the physical mixture were at 166.27 °C and 166.24 °C, respectively. Only the peaks of the excipients could be seen, and no ITZ peak was detected in the physical mixture, again probably due to the small quantity of ITZ in the mixture.

### 2.3. Stability Study

**Figure 4:**
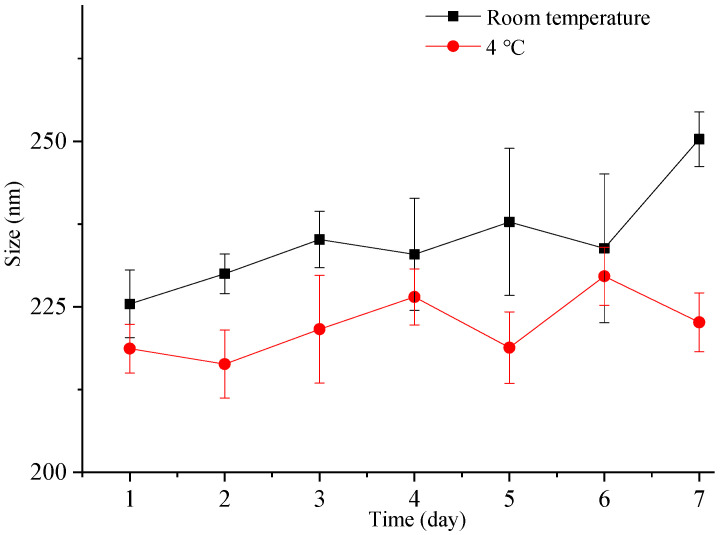
The particle size change in freshly prepared liquid ITZ-Nanos at room temperature and 4 °C.

Nanocrystals exhibit a higher total surface energy due to the large surface area associated with a small particle size, rendering them more susceptible to aggregation and other destabilization processes. Therefore, it is crucial to assess the particle size stability of nanocrystals. In this study, we investigated the particle size stability of the freshly prepared liquid ITZ-Nanos in a sealed container for one week at both room temperature and 4 °C. As depicted in Figure 4, the particle size of the ITZ-Nanos only increased by approximately 20 nm after one week at room temperature and exhibited a minimal increase at 4 °C. These findings demonstrate that the liquid ITZ-Nanos remained stable throughout the duration of our investigation. The stabilizer, CA, is an amphiphilic biomolecule with a steroid structure and four hydrophilic groups positioned on one side of the steroid plane: three hydroxyl groups (all in R positions) and a carboxylic acid group. Due to its molecular configuration, CA exhibits a large hydrophobic section area available for interaction with the substrate. Consequently, it can strongly adsorb onto the surface of nanoparticles while ensuring that the hydrophilic groups face outward, thereby providing an effective stabilizing barrier [23,31,32].

**Figure 5:**
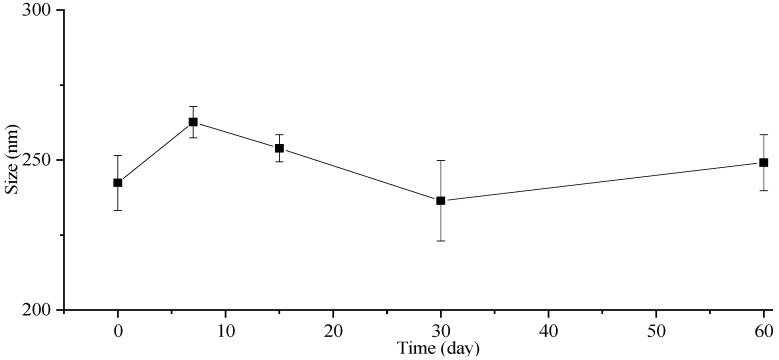
The particle size change in lyophilized ITZ-Nano powders within 60 days.

The stability of the particle size in the freeze-dried powders was also investigated, and the corresponding results are presented in Figure 5. The particle size of the freeze-dried ITZ-Nanos exhibited a negligible increase even after storage at room temperature for 7, 15, 30, and 60 days. Moreover, the particle size of the freeze-dried ITZ-Nanos remained comparable to that of the liquid ITZ-Nanos. Notably, mannitol incorporated into the formulation acts as an effective cryoprotectant [33], indicating the robustness of this formulation towards freeze-drying-induced stress. Consequently, the freeze-dried ITZ-Nano powders exhibit superior storage stability compared to their liquid counterparts and can be readily reconstituted when required.

### 2.4. In Vitro Dissolution under Supersaturating Conditions

**Figure 6:**
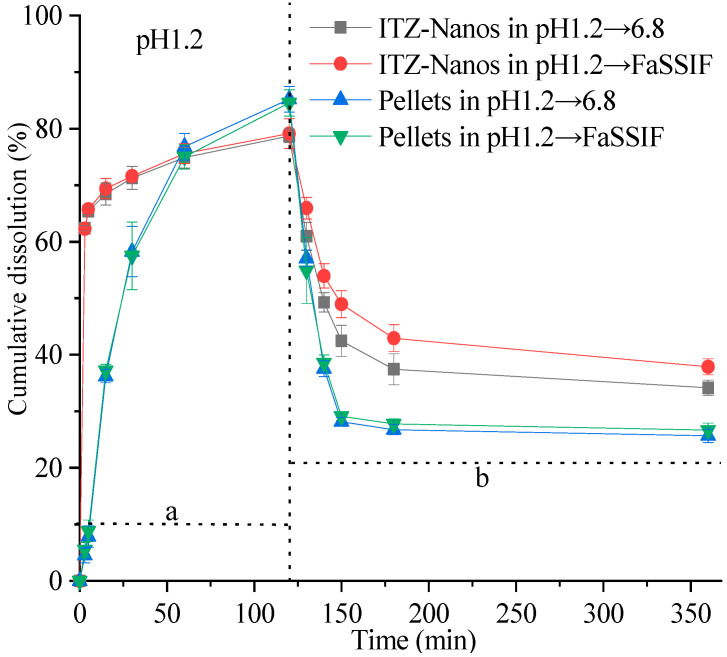
In vitro dissolution profiles of ITZ-Nanos and pellets under supersaturated conditions. a: dissolution profile corresponding to saturation dissolution at pH 1.2; b: dissolution profile corresponding to saturation dissolution with pH adjustment towards neutrality.

The supersaturation dissolution of the ITZ-Nanos at a pH of 1.2, followed by a pH of 6.8, was investigated and compared with the pellets in this study. Figure 6 illustrates the cumulative dissolution profiles of the ITZ-Nanos and pellets. Both formulations exhibited a rapid dissolution of ITZ, surpassing its equilibrium solubility within minutes at a pH of 1.2. Due to the significantly larger total surface area of the nanoparticles, the percentage of dissolved ITZ-Nanos increased rapidly to approximately 62% within the initial 3 min, followed by a slower increase to around 77% after 2 h in both media (pH 1.2 and pH 6.8). The release percentage for the commercial pellets showed a slower but steady increase, reaching about 82% after two hours. Both the ITZ-Nanos and pellets achieved substantial supersaturation. Upon adjusting the pH to 6.8, a significant increase in the overall volume of the medium was observed, as described in the Section 3. However, this led to a reduction in the percentage of ITZ dissolved for all the tested samples. This outcome was anticipated due to ITZ’s weak base nature and its decreased solubility with an increasing pH, resulting in crystallization from supersaturated solutions. After 3 h, approximately 40% of the ITZ remained dissolved in phosphate buffer saline (PBS) and 42% of it remained in fasting state simulated intestinal fluid (FaSSIF) for the ITZ-Nanos, whereas only about 27% remained dissolved for the pellets in both media. Notably, throughout the entire duration of up to 6 h, a considerably higher percentage of the ITZ-Nanos remained dissolved compared to the pellets. This difference could potentially be attributed to CA’s ability to inhibit drug crystallization within supersaturated solutions [34]. Although the excipients present in pellets have also been reported to possess crystallization inhibition effects [35,36], our study demonstrates that cholic acid exhibits superior efficacy.

Notably, the dissolution percentage of ITZ at a pH of 6.8 was higher in FaSSIF than in PBS. This observation could be attributed to the presence of bile salts and lecithin in FaSSIF, both of which act as surfactants. The synergistic effect between these surfactants and the stabiliser cholic acid in the ITZ-Nanos may contribute to inhibiting the crystallisation of ITZ.

### 2.5. Uptake of ITZ by Caco-2 Cells

**Figure 7:**
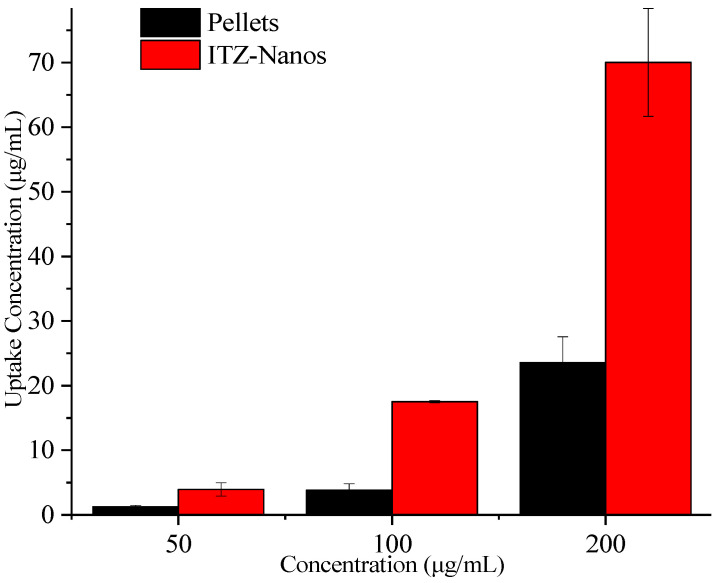
Uptake of ITZ in Caco-2 cells.

To assess the potential for enhanced absorption of nanocrystals, uptake experiments were conducted in Caco-2 cells for the ITZ-Nanos with pellets as the control. The results are presented in Figure 7. With an increase in the ITZ concentration, the total uptake of ITZ by the Caco-2 cells also increased. At all the tested concentrations, the ITZ-Nanos’ uptake was more than three times that of the pellets. This could be attributed to the superior dissolution profile of the ITZ-Nanos compared to the pellets, discussed earlier in the dissolution section, and phagocytosis on the part of the Caco-2 cells [37,38].

### 2.6. In Vivo Pharmacokinetics in Rats

**Figure 8:**
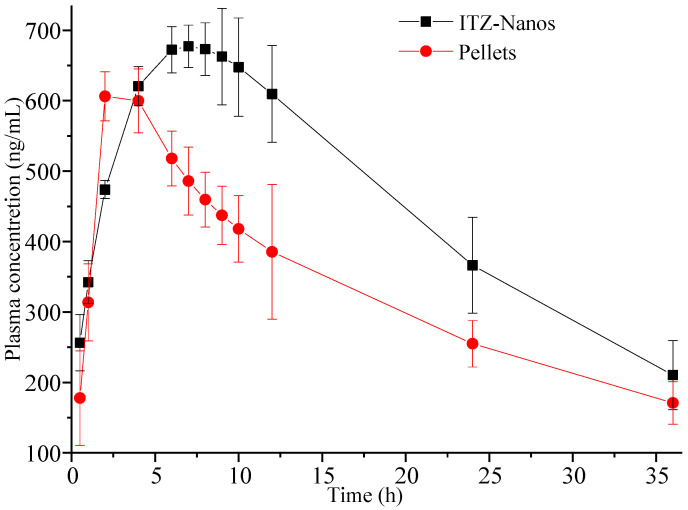
Plasma concentration–time profiles were determined in rats following oral administration of 15 mg/kg ITZ-Nanos and pellets (*n* = 5).

An in vivo pharmacokinetic study was conducted in rats. Figure 8 illustrates the mean plasma concentration–time (C-t) profiles for ITZ following oral administration at a single dose of 15 mg/kg [8,39], where the mean plasma concentration was a value fitted using DAS 2.0 software. It was obvious that the pharmacokinetic profile of the ITZ-Nano group was different in the peak concentration and peak time from the pellet group. The ITZ-Nano group demonstrated a peak around 8 h, while the pellets group showed a single peak at approximately 2 h.

The pharmacokinetic parameters of the two formulation groups were calculated and are presented in Table 2. The C_max_ values for the ITZ-Nano group were determined to be 742.49 ± 11.23 ng/mL. The large C_max_ can be attributed to the rapid dissolution at first and the subsequently continuous and slow dissolution of ITZ. Moreover, the AUC_(0–t)_ value for the ITZ-Nano group was found to be 1.33-fold higher than that of the pellet group, indicating the superior bioavailability of the ITZ-Nanos compared to the pellets.

Overall, our findings demonstrate that the in vivo absorption of the ITZ-Nanos was superior to that of the pellets. Previous studies have reported instances in which enhanced dissolution with nanocrystals did not translate into improved bioavailability, possibly due to the crystallization of dissolved ITZ when the pH approaches neutrality in the small intestine [23]. In contrast, the ITZ-Nanos developed in this study exhibited rapid dissolution at a low pH, leading to a supersaturated state that could be effectively maintained even after transitioning to neutral conditions in FaSSIF. Although efficient drug dissolution from formulations in gastric fluid is crucial for prompt oral absorption, sustaining high levels of drug supersaturation within the small intestine may also be pivotal in facilitating effective absorption [40,41].

## 3. Materials and Methods

Itraconazole was received from J&K Scientific Ltd. (Beijing, China). The Sporanox^®^ pellets (referred to as ‘pellets’ elsewhere) were the contents removed from the original commercial itraconazole capsules (Xian Janssen Pharmaceutical Ltd., Xi’an, China). Cholic acid (CA), FaSSIF, and morpholineethanesulfonic acid sodium salt were purchased from Shanghai Aladdin Biochemical Technology Co. Ltd. (Shanghai, China). Mannitol was obtained from the Tianjin branch of European Chemical Reagents Co. Ltd. (Tianjin, China). Nimodipine (99.8% purity) was obtained from Shanghai Source Leaf Biotechnology Co. Ltd. (Shanghai, China). All the other chemicals were analytical- or chromatography-grade.

### 3.1. Preparation of the ITZ-Nanos

The ITZ-Nanos were prepared using a combination of microprecipitation and high-pressure homogenization methods, as previously described [5]. Briefly, a suitable amount of CA and 50 mg of ITZ were completely dissolved in 2 mL of DMSO at 60 °C as the organic solvent phase. The antisolvent (50 mL water) was stirred at 1000 rpm using a magnetic stirrer (Gongyi City Yuhua Instrument Co., Ltd., Gongyi, China) at room temperature. The organic solvent phase was rapidly injected into the antisolvent to form a coarse suspension, which was continuously stirred for 20 min after the injection was completed. The obtained coarse suspension underwent a premilling process consisting of five cycles at 250 bar, followed by another five cycles at 500 bar, using a high-pressure homogenizer (model NS1001L2K, Niro Soavi S.p.A. Co, Parma, Italy). Finally, the suspension was homogenized for 20 cycles at a pressure of 1000 bar until a steady-state plateau in size was achieved [5]. To obtain the dry ITZ-Nano powder, a nanocrystal containing mannitol (10% *w*/*v*) was frozen at −20 °C for 12 h in a freezer and subsequently lyophilized using a Christ Alpha 1–2 LDplus freeze-drier (Martin Christ Gefriertrocknungsanlagen GmbH, Osterode, Germany) at −50 °C for 36 h. For analysis purposes, the dried ITZ-Nanos were reconstituted in deionized water.

### 3.2. Particle Size Analysis

The particle size, polydispersity index (PDI), and zeta potential of the ITZ-Nanos were determined using a Zetasizer Nano ZS90 (Malvern Instruments, Malvern, UK) based on dynamic light scattering before and after lyophilization. Each sample was measured three times, and the average value was calculated. Prior to measurement, the lyophilized ITZ-Nanos were reconstituted in deionized water.

### 3.3. Transmission Electron Microscopy

The particle morphology of the ITZ-Nanos and the ITZ bulk material was examined using a JEOL 2010 transmission electron microscope (TEM, JEOL Co., Tokyo, Japan). The freshly reconstituted ITZ-Nanos were deposited onto a carbon-coated copper grid and air-dried at room temperature. Images were captured under an acceleration voltage of 80 kV.

### 3.4. X-ray Diffraction (XRPD) Measurements

The diffraction pattern of the freeze-dried nanocrystals was investigated using an X-ray powder diffractometer (D8 ADVANCE, Bruker Co., Billerica, MA, USA). Cu Ka radiation (monochromator: graphite) was generated by setting the X-ray generator at 60 mA and 40 kV. The powder samples were placed between two PETP films in a rotating sample holder to obtain the X-ray diffraction patterns. The samples analyzed included physical mixtures of CA and mannitol (blank excipients), crude ITZ, and the physical mixture of CA, mannitol, and ITZ, as well as the lyophilized ITZ-Nanos. The spectra were collected with a step width of 0.05° and a 2θ range scanned from 5° to 50° at a rate of 4°/min.

### 3.5. Differential Scanning Calorimetry

The formulations were subjected to differential scanning calorimetry (DSC) using a TA-60WS thermal analyzer (Shimadzu, Kyoto, Japan). Briefly, the same samples used for the XRPD analysis were placed in aluminum pans and sealed with pinhole-pierced covers. An empty aluminum pan served as the reference. The samples were scanned under a nitrogen purge at a heating rate of 10 °C/min from 30 to 220 °C with a *N*_2_ flow rate of 20 mL/min.

### 3.6. Supersaturated Dissolution

The dissolution of the ITZ-Nanos was investigated using a ZRS-8G dissolution apparatus (Tianjin University Radio Factory, Tianjin, China) with attached paddles, as previously described [37,40]. In brief, ITZ-Nanos equivalent to 10 times the equilibrium solubility of ITZ (4.4 µg/mL at pH 1.2) were added to 10 mL of 0.1 mol/L HCl solution at 37 °C and stirred at a speed of 75 rpm. At predetermined time intervals, aliquots of 1 mL samples were collected from the dissolution medium and replaced with fresh medium of the same temperature (1 mL). After a duration of 120 min, the pH was adjusted to 6.8 by adding either 290 mL of a solution containing tribasic sodium phosphate (2.4 mM) or FaSSIF containing sodium taurocholate (3.10 mM), lecithin (0.77 mM), and the morpholineethanesulfonic acid sodium salt buffer component MES (42 mM). Aliquots of the samples (1.0 mL) were taken at time points of 130 min, 140 min, 150 min, 180 min, and 480 min. All the samples were filtered through a syringe filter with a pore size of 0.22 µm and analyzed using HPLC. All the experiments were performed three times.

### 3.7. Particle Size Stability

The freshly prepared liquid ITZ-Nanos were stored in sealed glass containers at 4 °C and room temperature (25 °C), respectively. Aliquots of 2 mL samples were withdrawn at 0, 1, 2, 3, 5, and 7 days for particle size measurement. The freeze-dried ITZ-Nanos were stored in sealed glass containers at room temperature. Approximately 6 mg of the powder was removed at intervals of 0, 7, 15, 30, and 60 days and reconstituted in deionized water (ratio: 2 mg to 1 mL). The particle size of the reconstituted samples was immediately measured after reconstitution. All the measurements were performed three times.

### 3.8. In Vitro Uptake across the Caco-2 Monolayer

#### 3.8.1. Caco-2 Culture

The Caco-2 cells were obtained from the American Tissue Culture Collection (Rockville, MD, USA) with a passage number ranging from 50 to 70. These cells were routinely cultivated in DMEM (4, 500 mg/L glucose) supplemented with 10% FBS, 1% nonessential amino acids, 100 U/mL penicillin, 100 µg/mL streptomycin, and 1.0 mmol/L sodium pyruvate under a humidified atmosphere at 37 °C with 5% CO_2_. The growth medium was refreshed every 2–3 days after incubation.

#### 3.8.2. Uptake of ITZ by the Caco-2 Cells

The Caco-2 cells were seeded into 24-well plastic cluster trays at a density of 1 × 10^5^ cells/cm^2^. The cells were fed every two days during the first week and daily thereafter. After 15 days, the cell monolayers were washed twice with fresh Hank’s Balanced Salt Solution (HBSS) and incubated in HBSS for 30 min. Subsequently, the cells were incubated with ITZ-Nanos or pellets diluted in non-complete culture medium at concentrations of approximately 50, 100, and 200 µg/mL at a temperature of 37 °C for a duration of 30 min. Following this incubation period, the cells were washed three times with ice-cold HBSS solution (1.0 mL) and supplemented with an additional volume of 0.3 mL of NaCl (0.9%). The cells were then scraped into an Eppendorf tube containing added water (0.3 mL). To obtain cell lysates, ultrasonication was employed using a JY92-IIDN ultrasonic cell crusher (from Ningbo Xinzhi Biotechnology Co., Ltd., Ningbo, China). These lysates underwent subsequent centrifugation at a force of 15,000× *g* for ten minutes to separate the supernatants, which were subsequently analyzed using HPLC. The cellular uptake was normalized to the microgram protein content determined using a BCA protein assay kit, where bovine serum albumin served as the standard reference compound. Each sample was subjected to analysis three times.

### 3.9. The In Vivo Pharmacokinetic Study in Rats

The animal experiments conducted in this study were approved by the Committee of Ethics of Animal Experimentation at Henan University, China (No. HUSOM2016-066). Ten adult male Wistar rats weighing approximately 200 to 230 g were housed under controlled conditions (temperature: 25 °C; relative humidity: 45%) for a minimum of one week prior to the pharmacokinetic study. The rats underwent a fasting period of 12 h with ad libitum access to water before the experiments and were randomly and equally divided into two groups. The ITZ-Nanos and pellets were orally administered using an intragastric needle at a dose of 15 mg/kg [8]. Serial blood samples (approximately 0.5 mL) were collected at various time points (0.5, 1, 2, 4, 6, 7, 8, 9, 10, 12, 24, and 36 h post-dose) from the post-orbital plexus using heparinized tubes. After immediate centrifugation at a speed of 3000 r/min for 10 min, the plasma samples were collected and stored at −20 °C until the analysis.

### 3.10. HPLC Analysis

The high-performance liquid chromatography (HPLC) analysis of the ITZ was conducted using a Waters 2695 HPLC system (Waters Corporation, Milford, MA, USA) equipped with an autosampler and a variable-wavelength UV detector. A reverse-phase C-18 column (5 µm, 250 mm × 4.6 mm, Thermo Syncronis, Waltham, MA, USA) was employed, and the mobile phase consisting of methanol/water at a ratio of 85:15 *v*/*v* was maintained at a flow rate of 1.0 mL/min at 25 °C. The detection wavelength was set at 263 nm, and a sample volume of 20 µL was injected into the column for subsequent analysis. Calibration curves ranging from concentrations of 0.1 to 100 µg/mL were prepared by employing ITZ standard solutions dissolved in methanol [5].

For the analysis of the plasma samples, a protein precipitation procedure with methanol was used with nimodipine added as the internal standard. Briefly, 100 μL of methanol and 200 μL of nimodipine solution (2 μg/mL) were added to 100 μL of plasma and vortexed for 3 min. The suspension was sonicated for 30 s and subsequently centrifuged at 16,000× *g* for 10 min. The supernatant was collected and dried under a nitrogen flow stream. Then, 100 μL of methanol was added to the residue and vortexed for 3 min. The sample was sonicated for 30 s and centrifuged at 16,000× *g* for 10 min. Subsequently, 20 μL of the supernatant was analyzed using HPLC.

### 3.11. Data Analysis

The pharmacokinetic parameters were calculated using the DAS 2.0 pharmacokinetic program (Mathematical Pharmacology Professional Committee of China, Shanghai, China). Statistical differences were analyzed using a two-tailed Student’s *t*-test in SPSS 12.0.

## 4. Conclusions

In this study, ITZ-Nanos with CA as a stabilizer were prepared using a microprecipitation–high-pressure homogenization method. A drug-to-CA ratio of 1:1 was shown to have the best stabilizing effect. The ITZ-Nanos can dissolve quickly to achieve a high degree of supersaturation at a low pH. They can maintain a good level of supersaturation after the pH is changed to neutral. Compared with the pellets, the ITZ-Nanos also showed improved uptake by the Caco-2 cells and enhanced bioavailability rates. The in vivo pharmacokinetics test in this study suggested that the advantage of the ITZ-Nanos stabilized using endogenous small-molecule in vitro dissolution enhancement could be presented as improved oral availability compared to pellets. The above results indicate that CA is a promising stabilizer for improving the oral bioavailability of the ITZ-Nanos and potentially for other insoluble drugs as well.

## Figures and Tables

**Table 1:** Particle sizes of nanocrystals with different drug/stabilizer ratios.

Drug/Stabilizer Ratio	Size (nm)	PDI	Zeta Potential (mV)
2:1	273 ± 25.2	0.288 ± 0.027	−15.2 ± 1.8
1:1	225.7 ± 10.3	0.189 ± 0.023	−28.1 ± 2.3
1:2	262.1 ± 23.5	0.268 ± 0.016	−23.3 ± 2.7

**Table 2:** Pharmacokinetic parameters in rats after oral administration of 15 mg/kg ITZ-Nanos and pellets (*n* = 5).

Parameters	Unit	ITZ-Nanos	Pellets
AUC_(0–t)_	μg·L^−1^·h^−1^	16,065.68 ± 1514.1 *	12,084.46 ± 1186.3
AUC_(0–∞)_	μg·L^−1^·h^−1^	20,863.45 ± 2917.5	17,305.51 ± 2035.7
C_max_	μg·L^−1^	742.49 ± 11.23 **	607.35 ± 23.12
T_max_	h	8.00 ± 0.57 **	2.00 ± 0.87
MAT	h	24.69 ± 1.86	30.65 ± 2.36

The single and double asterisks indicate statistical significance at *p* < 0.05 and *p* < 0.001, respectively.

## Data Availability

The dataset is available on request from the authors.
